# Application of combined model of stepwise regression analysis and artificial neural network in data calibration of miniature air quality detector

**DOI:** 10.1038/s41598-021-82871-4

**Published:** 2021-02-05

**Authors:** Bing Liu, Qingbo Zhao, Yueqiang Jin, Jiayu Shen, Chaoyang Li

**Affiliations:** 1 Public Foundational Courses Department, Nanjing Vocational University of Industry Technology, Nanjing, 210023 China; 2 Public Foundational Courses Department, Sanmenxia Polytechnic, Sanmenxia, 472000 China; 3 College of Management, Henan University of Technology, Zhengzhou, 450001 China

**Keywords:** Atmospheric science, Climate change

## Abstract

In this paper, six types of air pollutant concentrations are taken as the research object, and the data monitored by the micro air quality detector are calibrated by the national control point measurement data. We use correlation analysis to find out the main factors affecting air quality, and then build a stepwise regression model for six types of pollutants based on 8 months of data. Taking the stepwise regression fitting value and the data monitored by the miniature air quality detector as input variables, combined with the multilayer perceptron neural network, the SRA-MLP model was obtained to correct the pollutant data. We compared the stepwise regression model, the standard multilayer perceptron neural network and the SRA-MLP model by three indicators. Whether it is root mean square error, average absolute error or average relative error, SRA-MLP model is the best model. Using the SRA-MLP model to correct the data can increase the accuracy of the self-built point data by 42.5% to 86.5%. The SRA-MLP model has excellent prediction effects on both the training set and the test set, indicating that it has good generalization ability. This model plays a positive role in scientific arrangement and promotion of miniature air quality detectors. It can be applied not only to air quality monitoring, but also to the monitoring of other environmental indicators.

## Introduction

Air quality is becoming more and more important. It affects both the natural environment and human health. The relationship between cardiovascular disease, lung cancer, respiratory system disease and air pollution has been confirmed by some documents^1–3^. Real-time monitoring of the concentration of major pollutants (''two dusts and four gases" includes PM2.5, PM10, CO, NO_2_, SO_2_, O_3_) in the atmosphere is becoming more and more necessary for relevant national departments. The national monitoring and control station (national control point) can measure the concentration of pollutants, and the "two dust and four gases" monitoring data of the national control point (ncp) is considered accurate. However, due to cost issues, the number of national control points is small, and it is difficult to meet the requirements for real-time monitoring of air quality. Some miniature air quality detectors (self-built points) are gridded and deployed in some areas. They can realize real-time monitoring of air quality, and can also monitor other meteorological parameters (temperature, humidity, wind speed, pressure and precipitation) in the area. Since the electrochemical sensor used in the self-built point (sbp) will be interfered by external factors, it will cause measurement errors^4^. We need to use the national control point data to calibrate the self-built point data.

Mechanism models based on atmospheric chemical analysis and statistical models based on machine learning are often used to predict the concentration of pollutants. The former uses meteorological principles and mathematical methods to simulate the chemical and physical processes of pollutants to realize the prediction of pollutant concentration^5,6^. The latter uses statistical methods to analyze the collected pollutant data and uses mathematical algorithms to model the relationship between variables. For the research based on machine learning models, the main algorithms are artificial neural networks^7–9^, multiple linear regression^10–12^, hidden Markov models^13,14^, random forest models^15–17^, and support Vector machine^18–20^ and so on.

Artificial neural network (ANN) is an information processing system that simulates human brain thinking and reasoning. It has been a research hotspot in the field of artificial intelligence since the 1980s, and has made certain progress in various research fields. Its advantage is that it has strong nonlinear fitting ability, can map arbitrarily complex nonlinear relationships. Artificial neural networks have strong associative storage capabilities, robustness, non-linear mapping capabilities, and autonomous learning capabilities. However, it turns all the characteristics of the problem into numbers and turns all reasoning into numerical calculations^21–23^, so it has no ability to explain its reasoning process and reasoning process. As a mature method for solving linear problems, multiple linear regression (MLR) has been widely used in various fields. Its advantage is that it is more convenient and simple when analyzing a multi-factor model. If the data used is the same as the model, the calculation result is unique, and each regression coefficient in the model is better explained^11,24,25^. However, multiple linear regression models have strict requirements on independent variable selection and error terms, and multiple linear regression methods are also greatly restricted in solving nonlinear problems.

Artificial neural networks and multiple linear regression models are widely used in air quality prediction models. The two-step calibration method of multiple linear regression and machine learning was used by Elangasinghe et al. to correct the NO_2_ concentration measured by the sensor. They compared different machine learning methods through 5 evaluation indicators and gave the best model^7^. Artificial neural networks are used by Reich, S. L. et al. to identify pollution sources in the air. They chose to use a three-layer feedforward ANN trained by the backpropagation algorithm and successfully repaired some of the data in the model^9^.Spinelle, L. et al. compared linear/multilinear regression and supervised learning techniques, and carried out on-site calibration of NO, CO and CO_2_ pollutant sensors^10^. However, both linear regression and artificial neural network have shortcomings in air quality prediction models^26^. In this paper, by combining the prediction effects of the two methods in the air quality forecast model data, a calibration model of the main pollutants in the air is given to improve the interpretability and accuracy of the air quality calibration model.

## Material and methods

### Data source and preprocessing

This article selects 2019 Chinese college students' mathematical modeling D problem data. It provides hourly data of a national control point from November 14, 2018 to June 11, 2019. It also provides a self-built point data corresponding to the national control point (corresponds to the national control point time and the interval is within 5 min). Before conducting exploratory analysis on the data of national control points and self-built points, the data is pre-processed. First, delete the data that the self-built point and the national control point cannot correspond to and the data that is obviously abnormal. Second, the various data within each hour of the self-built point are classified and aggregated and averaged to correspond to the hourly data of the national control point. After data preprocessing, a total of 4135 sets of data were obtained as research objects^27^. Table 1 shows the range, mean, and standard deviation of each variable.

**Table 1:** Descriptive statistics of air quality variables from data from national control points and self-built points.

Input variable	Ranges	Mean	Standard deviation
PM2.5/(μg/m^3^)	1–216.883	64.127	37.328
PM10/(μg/m^3^)	2–443.25	102.391	65.267
CO/(μg/m^3^)	0.05–3.895	0.863	0.452
NO_2_/(μg/m^3^)	0.947–157.136	45.209	28.403
SO_2_/(μg/m^3^)	1–651.3	19.397	18.723
O_3_/(μg/m^3^)	0.579–259	61.586	40.941
Wind speed/(m/s)	0.133–2.387	0.7	0.346
Pressure/(Pa)	996.871–1039.8	1018.8	8.889
Precipitation/(mm/m^2^)	0–312.1	132.084	87.004
Temperature/(℃)	− 3.882 to 37.944	11.882	8.603
Humidity/(rh%)	10.667–100	68.903	21.931

### Data exploratory analysis

The establishment of statistical models usually starts with exploratory analysis of the data^11,28,29^. Based on the national control point data, the “two dusts and four gases” concentration data measured at the self-built points are corrected in this paper. In order to more intuitively reflect the difference between the national control point and the self-built point data, we calculated the daily average value of the preprocessed 4135 sets of data and compared these pollutant concentration data.

**Figure 1:**
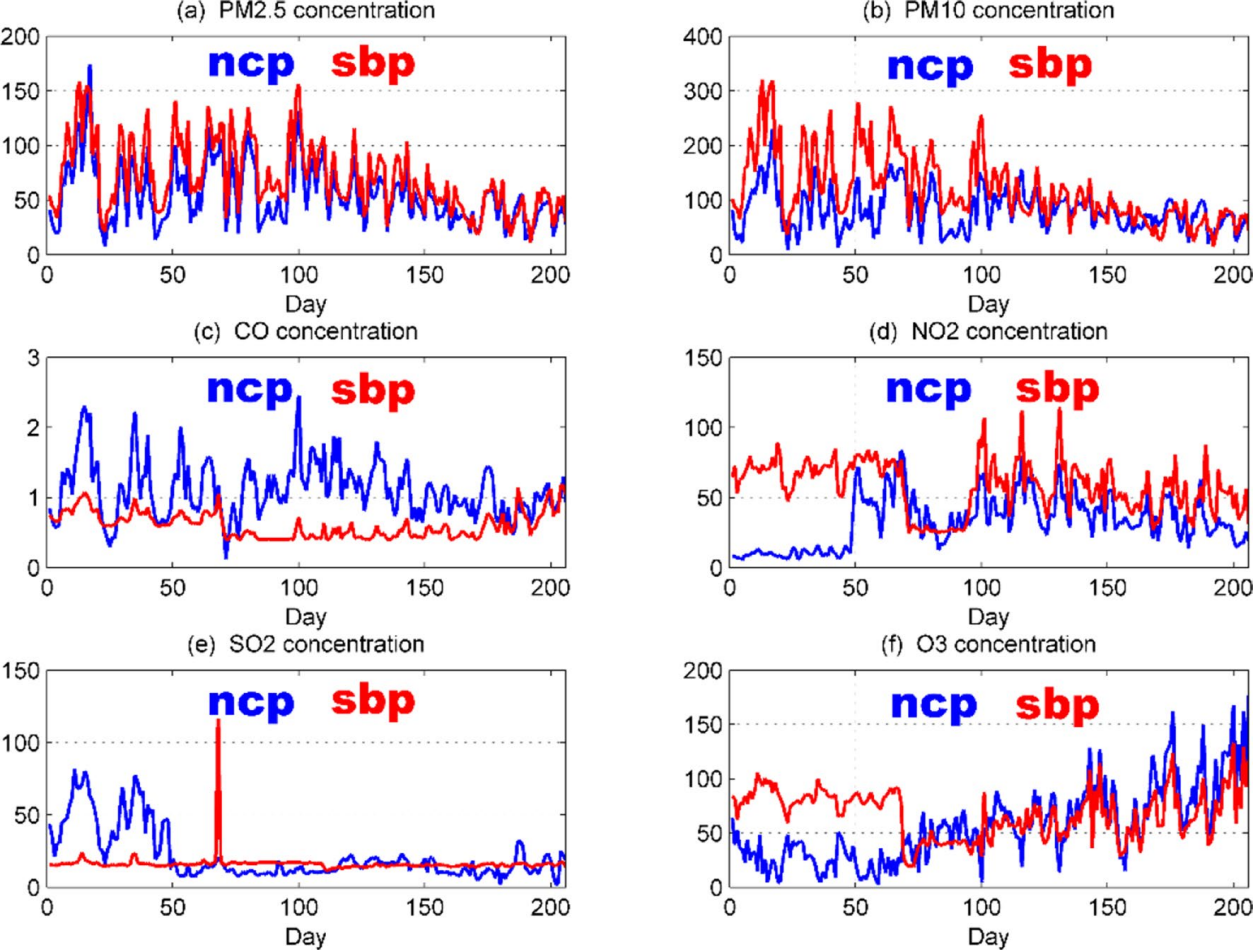
Comparison of daily average data of six types of pollutants at national control points and self-built points.

**Figure 2:**
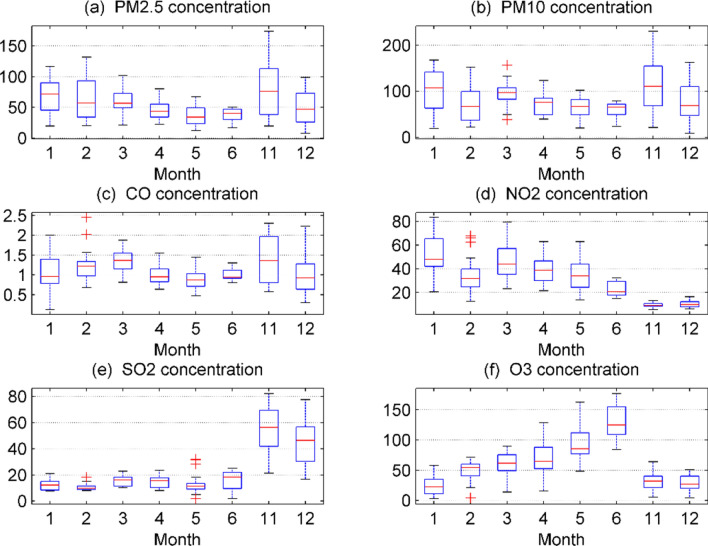
Comparison of monthly average data of six types of pollutants at national control points and self-built points. Figures are generated using Matlab (Version R2016a, https://www.mat-hworks.com/) (software).

In Fig. 1, the blue curve indicates the national control point measurement value, and the red curve indicates the self-built point measurement value. It can be seen that the measurement data of the “two dusts and four gases” concentration national control point and the self-built point are generally consistent, but there is a certain deviation between the two. The deviation between the two in the previous period is significantly larger, which may be caused by the season or the zero drift of the measuring instrument. As the PM2.5, PM10, and O_3_ concentrations change significantly over time, we draw a box-line diagram^10^ of the monthly changes in the concentration of the “two dusts and four gases” national control points as shown in Fig. 2.

It can be seen from Fig. 2: The average PM2.5, PM10, CO, and SO2 concentrations are highest in November, the average NO_2_ concentration is highest in January, and the average O_3_ concentration is highest in June. The average PM2.5, CO, and SO_2_ concentrations are lowest in May, the average PM10 concentration is lowest in June, the average NO_2_ concentration is lowest in November, and the average O_3_ concentration is lowest in December. The concentration of "two dusts and four gases" varies significantly in different months, so time is an important factor affecting the concentration of "two dusts and four gases".

### Correlation analysis

**Figure 3:**
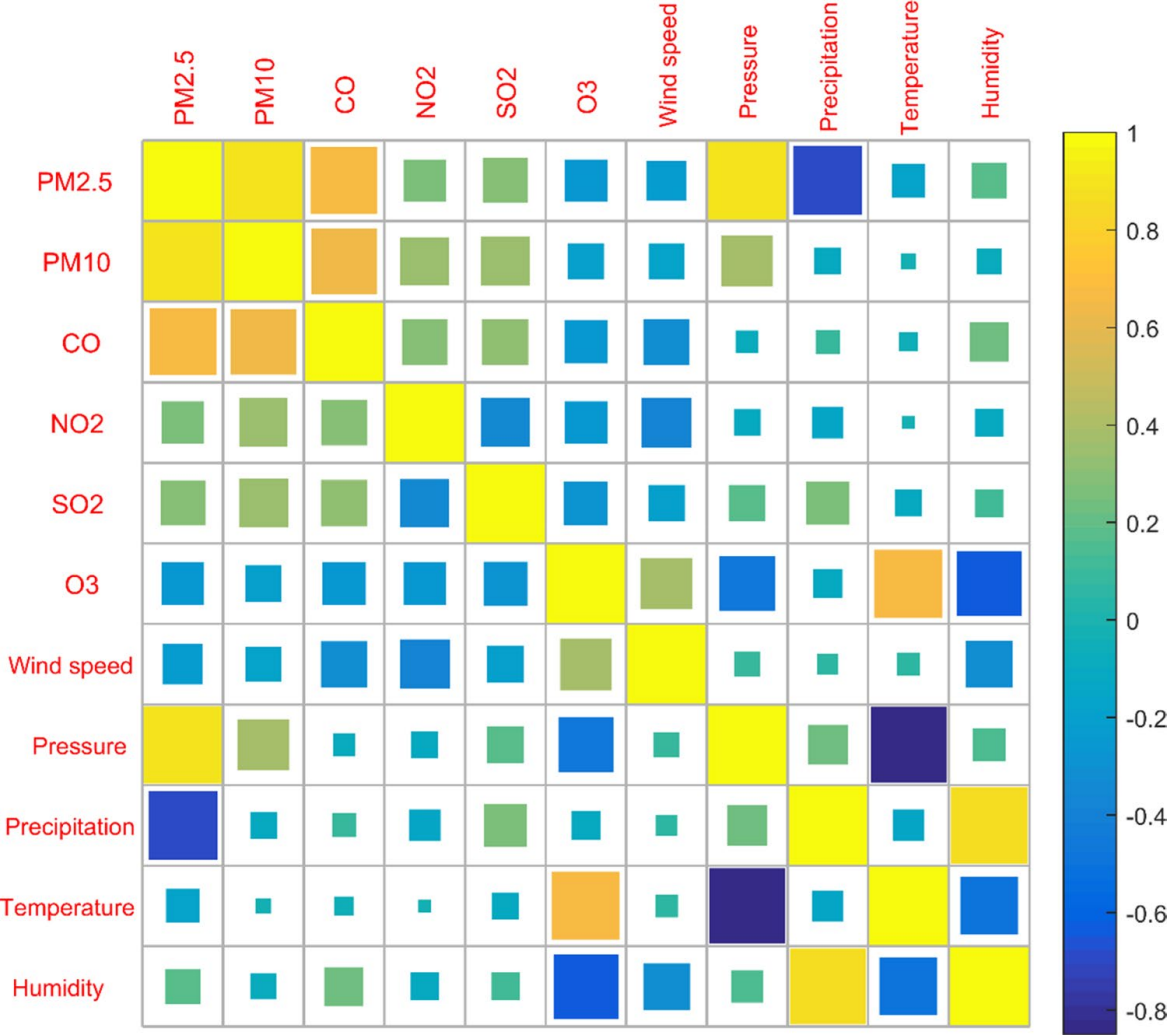
Correlation coefficient matrix color block diagram between six types of air pollutant concentrations and climate.

The quality of air is judged based on the concentration of pollutants in the air^1^. There are many factors that affect air quality, and they affect each other. In order to determine the correlation between the "two dusts and four gases" concentration and the five climate factors^30^, we use Eq. (1) to find the Pearson correlation coefficient between them, as shown in Table 2. It can be seen that, except for NO_2_ concentration and temperature, all other variables have significant correlations with each other, indicating that the factors affecting the concentration of each pollutant are very complex. The correlation coefficient between PM2.5 concentration and PM10 concentration is as high as 0.89, indicating a high positive correlation between the two, and the correlation coefficient between temperature and air pressure is -0.85, which indicates that the higher the temperature, the lower the pressure. Figure 3 is a matrix color block diagram between the concentration of "two dusts and four gases" and five climatic factors, which visually shows the correlation coefficients between the variables. The size of the matrix color block represents the absolute value of the correlation coefficient. As the color becomes lighter, the value of the correlation coefficient gradually increases.

**Table 2:** Pearson linear correlation coefficients between six types of air pollutant concentrations and climate (band * indicates significant correlation at a significant level of 0.05).

	PM2.5	PM10	CO	NO_2_	SO_2_	O_3_	Wind speed	Pressure	Precipitation	Temperature	Humidity
PM2.5	1.00	0.89*	0.66*	0.26*	0.29*	− 0.26*	− 0.23*	0.89*	− 0.70*	− 0.16*	0.18*
PM10		1.00	0.63*	0.34*	0.35*	− 0.19*	− 0.18*	0.38*	− 0.10*	− 0.03*	− 0.09*
CO			1.00	0.30*	0.31*	− 0.27*	− 0.31*	− 0.07*	0.08*	− 0.05*	0.22*
NO_2_				1.00	− 0.34*	− 0.26*	− 0.36*	− 0.10*	− 0.14*	− 0.02	− 0.11*
SO_2_					1.00	− 0.28*	− 0.19*	0.19*	0.27*	− 0.10*	0.11*
O_3_						1.00	0.39*	− 0.45*	− 0.12*	0.68*	− 0.62*
Wind speed							1.00	0.09*	0.06*	0.07*	− 0.32*
Pressure								1.00	0.23*	− 0.85*	0.15*
Precipitation									1.00	− 0.14*	0.86*
Temperature										1.00	− 0.49*
Humidity											1.00

1 $$r=\frac{\sum_{i=1}^{n}({x}_{i}-\overline{x})({y}_{i}-\overline{y})}{\sqrt{{\sum_{i=1}^{n}({x}_{i}-\overline{x})}^{2}}\bullet \sqrt{{\sum_{i=1}^{n}({y}_{i}-\overline{y})}^{2}}}$$

## Establishment of sensor calibration model

### Introduction to basic principles

Artificial neural network is one of the most commonly used methods to predict the concentration of atmospheric pollutants. It has the ability to approximate any non-linear mapping through learning. It has a wide application prospect in the prediction of non-linear systems. The working principle of artificial neural network prediction is mainly divided into two steps: first, use the training samples to design and train the network to obtain prediction rules; then predict the test samples according to the obtained rules to verify its reliability with the accuracy of the test results. The main advantage of artificial neural network algorithms is their strong adaptability to training samples. It has a strong ability to process uncertain information. It can still work normally for the presence of noisy or non-linear data. Artificial neural network has strong robustness, memory ability, non-linear mapping ability and strong self-learning ability in training. It can quickly get prediction results for complex prediction problems. After consulting relevant literature, the most commonly used model in the research and application of neural networks are multilayer perceptron neural network^31–33^.

**Figure 4:**
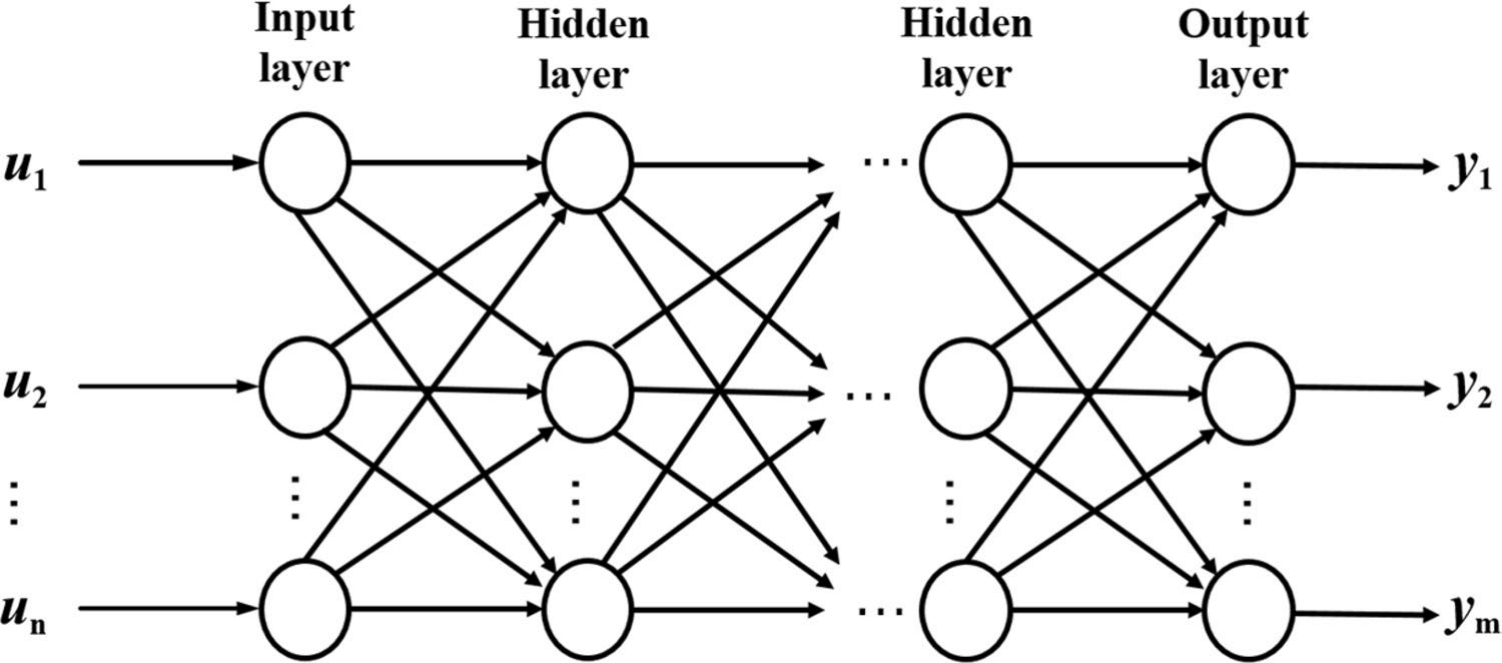
Multilayer perceptron neural network structure.

Multilayer Perceptron (MLP) neural network is a unidirectional propagation multilayer feedforward network structure based on error back propagation algorithm. As shown in Fig. 4: its structure can be divided into three layers, namely the input layer, the hidden layer and the output layer. Each layer of it consists of multiple nodes, and each layer can be passed to the next layer until the output layer. Except for the input nodes, each node is a neuron with a non-linear activation function. Equation (2) is its output, ${\upomega }_{nj}$ is the node weight, and ${b}_{jk}$ is the deviation.

2 $${o}_{k}=\sum_{j}{\upomega }_{nj}{x}_{n}+{b}_{jk}$$

3 $$J(\upomega ,b;x,y)=\frac{1}{2}{\Vert {o}_{\upomega ,b(x)}-y\Vert }^{2}$$

MLP is a typical supervised learning algorithm, and its loss function is defined as Eq. (3). ${o}_{\upomega ,b(x)}$ is the output value of MLP, and y is the actual value. In this paper, the parameters are adjusted by the conjugate gradient method to minimize the loss function. The conjugate gradient method calculation formulas are Eqs. (4) and (5). The hidden layer in the MLP neural network model can be single or several. However, as long as the number of neuron nodes in the hidden layer is appropriately adjusted, a single hidden layer neural network can approximate any nonlinear function^34,35^. Therefore, a single hidden layer can meet most engineering needs. In the process of using SPSS software for auxiliary calculation, the number of hidden layer neurons can be automatically calculated by SPSS, and the relatively optimal number of neurons that is most suitable for this model is given.

4 $$S\left(n+1\right)=-g\left(n+1\right)+\beta (n+1)\times S(n)$$

5 $$\beta \left(n\right)=\frac{(-g{(n+1))}^{T}\times (g\left(n\right)-g\left(n+1\right))}{g{\left(n\right)}^{T}\times g\left(n\right)}$$

The concentration of "two dusts and four gases" is affected by various factors such as various climatic factors and other pollutant concentrations, as well as the sensor's own range drift. The simple regression model can only describe the linear effect of each variable on the concentration of pollutants. The appropriate weighted average of the model by the neural network, and introducing other non-linear effects into the model, can effectively improve the prediction accuracy of the model and improve the correction effect of the self-built point pollutant concentration.

**Figure 5:**
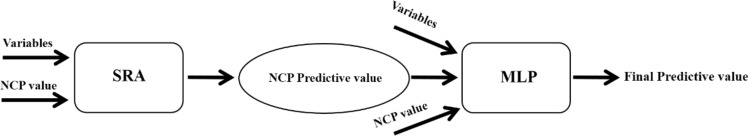
The flux diagram of the regression process.

In this paper, we will build a combination model of stepwise regression analysis (SRA) and artificial neural network, called SRA-MLR model. Firstly, a stepwise regression model is established through the influence of various factors on the concentration of pollutants, and the stepwise regression model is used to give the fitted value of each pollutant at the corresponding moment. Then the SRA-MLP neural network model is established by taking the fitted value and other data and time measured by the self-built point as input values and the national control point data as output values. The process of building the model is shown in Fig. 5.

### Stepwise regression model construction

We want to establish a multiple regression model with the pollutant concentration at the national control point as the dependent variable and the observation data from the self-built point as the independent variable. The key to establishing a multiple regression model is the choice of independent variables. If too few independent variables are selected, it is easy to miss key variables and the regression effect is not ideal. Too many independent variables are introduced into the model, which is prone to multicollinearity problems, which makes the model very unstable, and even problems such as inversion of sign. Commonly used independent variable selection methods are forward, backward, stepwise method. We use stepwise regression to build the model. The variables introduced in the model and their regression coefficients are given in Table 3.

**Table 3:** Stepwise regression model and model test of six types of air pollutant concentrations. In the model, the dependent variable is the concentration of the six pollutants at the national control point, and the independent variable is the variable and time monitored by the self-built point (– represents the variables eliminated in the model).

Independent variable	PM2.5	PM10	CO	NO_2_	SO_2_	O_3_
Constant	451.574	1401.748	23.215	792.815	32.027	− 1216.497
PM2.5/(μg/m^3^)	0.792	0.781	0.007	0.330	0.040	0.770
PM10/(μg/m^3^)	0.026	0.101	–	− 0.131	–	− 0.455
CO/(μg/m^3^)	9.3	28.346	0.473	7.372	17.857	–
NO_2_/(μg/m^3^)	0.079	0.353	0.002	0.441	0.030	− 0.572
SO_2_/(μg/m^3^)	—	0.088	–	–	− 0.033	0.043
O_3_/(μg/m^3^)	–	–	0.001	–	–	0.624
Wind speed/(m/s)	–	–	− 0.113	− 13.396	− 10.228	19.374
Pressure /(Pa)	− 0.428	− 1.297	− 0.022	− 0.730	–	1.186
Precipitation /( mm/m^2^)	− 0.031	− 0.077	3.28E−4	− 0.044	0.030	–
Temperature /(℃)	− 0.195	− 1.105	− 0.023	− 2.233	0.915	2.018
Humidity /( rh%)	− 0.342	− 1.146	− 0.003	− 0.520	− 0.092	− 0.113
Time/ (hour)	–	− 0.002	4.96E−5	0.011	− 0.013	0.011
F value	5100.060	1765.767	428.024	627.432	577.016	1863.809
R^2^	0.908	0.811	0.509	0.603	0.557	0.819

The F-test p-values in the six types of pollutant regression models are all less than 0.01, indicating that at a significant level of 0.01, the variables introduced into the model as a whole have a significant effect on the concentration of pollutants. The t-test p-value of each independent variable introduced into the model is less than 0.05, indicating that at a significant level of 0.05, each independent variable introduced into the model has a significant effect on the concentration of pollutants. The coefficient of determination in the PM2.5 concentration model is 0.908, indicating that the fitting effect is very good; the coefficients of determination in the PM10 and O_3_ concentration models are all greater than 0.8, indicating that the fitting effect is good; the coefficients of determination in the CO, NO_2_, and SO_2_ concentration models are all greater than 0.5, indicating that the fitting effect is acceptable.

### SRA-MLP model construction

The miniature air quality detector can not only implement grid-based monitoring of the air quality in the area, but also monitor meteorological parameters such as temperature, humidity, wind speed, air pressure, and precipitation. The fitting values of the air pollutant concentrations of the stepwise regression model and the data from the self-built points were used as covariate factors in the MLP model, and the air pollutant concentrations at the national control point were used as the dependent variables. We use SPSS 20.0 to fit the non-linear relationship between the covariate factors and the dependent variables.

In the MLP neural network, it is particularly important to choose the number of hidden layers and the number of neurons in each layer. In a small data set, too many hidden layers will not only make the model more complicated, but also lead to overfitting of the model and poor model generalization ability. Therefore, in small data sets, one or two hidden layers MLP neural network is generally used for modeling. We establish one hidden layer and two hidden layers MLP models for six types of pollutants, and choose the model with less error as the final prediction model of the pollutants. In the modeling process, 4135 samples are randomly assigned as training samples, test samples, and holdout samples, and the allocation ratio is 7:2:1, and the activation functions of the input layer and output layer adopt hyperbolic tangent function and identity function respectively. The batch is selected as the type of training, and scaled conjugate gradient is selected as the optimization algorithm. The software automatically calculates the number of units in the hidden layer and finally obtains SRA-MLP model^36^.

6 $$RMSE=\sqrt{\frac{1}{n}\sum_{t=1}^{n}{({y}_{t}-{w}_{t})}^{2}}$$

7 $$MAE=\frac{1}{n}\sum_{t=1}^{n}\left|{y}_{t}-{w}_{t}\right|$$

8 $$MAPE=\frac{1}{n}\sum_{t=1}^{n}\left|\frac{{y}_{t}-{w}_{t}}{{y}_{t}}\right|$$

**Figure 6:**
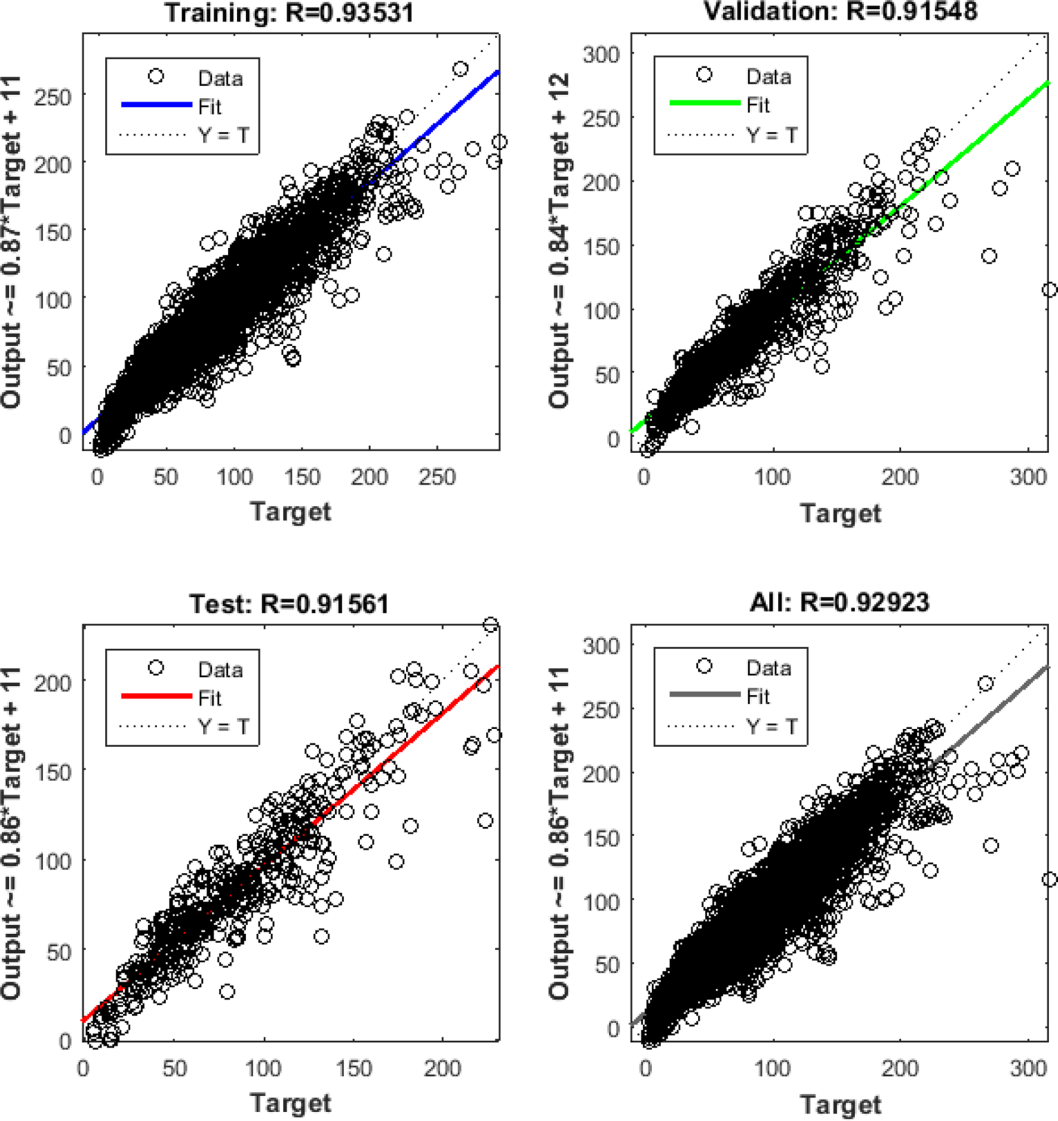
The prediction effect of PM10’s SRA-MLP model on the training set, validation set and test set.

This article uses root mean square error(Eq. 6), mean absolute error(Eq. 7), and mean absolute percent error(Eq. 8) to determine the final hidden layer number. The specific results are shown in Table 4. It can be seen that in NO_2_ and O_3_ prediction models, the two hidden layers MLP model performs better, so NO_2_ and O_3_ finally choose the two hidden layers SRA-MLP model. The numbers of neurons in the first and second layers of the NO_2_ prediction model are 8 and 6, and the numbers of neurons in the first and second layers of the O_3_ prediction model are 8 and 6. PM2.5, PM10, CO and SO_2_ finally choose one hidden layer SRA-MLP model, and the number of their hidden layer neurons are 7, 6, 5, and 8. The effect of our randomly selected PM10 prediction model is shown in Fig. 6. It can be seen that the prediction effect of the SRA-MLP model is very good whether it is the training set, validation set or test set.

**Table 4:** Comparison of neural network errors between one hidden layer and two hidden layers. The first three columns are the model errors of one hidden layer of six types of pollutants, and the last three columns are the model errors of two hidden layers of six types of pollutants.

Input variable	RMSE1	MAE1	MAPE1	RMSE2	MAE2	MAPE2
PM2.5	9.311	6.591	0.163	9.367	6.576	0.152
PM10	16.980	11.907	0.205	18.362	12.943	0.205
CO	0.222	0.165	0.183	0.235	0.173	0.204
NO_2_	10.627	7.720	0.320	10.331	7.215	0.287
SO_2_	7.811	5.270	0.394	8.089	5.087	0.351
O_3_	16.469	12.529	0.908	15.629	11.513	0.635

## Discussion

In the air quality prediction problem, stepwise regression models, MLP and SRA-MLP models can fit the data of self-built points. We can verify each model by the error between the model prediction value and the national control point data. Obviously, which model has a smaller error between the predicted value and the national control point value, which model is better. This article uses root mean square error, mean absolute error, and mean absolute percent error to evaluate the model^30^. The specific results are shown in Tables 5, 6 and 7.

**Table 5:** RMSE of six types of air pollutant concentrations between self-built points, model forecast values and national control point.

Input variable	Self-built points	SRA	MLP	SRA-MLP
PM2.5	22.436	10.147	10.226	9.311
PM10	66.263	20.004	19.149	16.980
CO	0.679	0.343	0.265	0.222
NO_2_	37.183	15.332	12.126	10.331
SO_2_	26.24	13.287	9.235	7.811
O_3_	45.673	20.429	17.695	15.629

**Table 6:** MAE of six types of air pollutant concentrations between self-built points, model forecast values and national control point.

Input variable	Self-built points	SRA	MLP	SRA-MLP
PM2.5	18.181	7.027	7.417	6.591
PM10	50.151	13.677	13.148	11.907
CO	0.549	0.261	0.196	0.165
NO_2_	29.838	11.61	8.787	7.215
SO_2_	12.867	9.394	6.093	5.270
O_3_	36.63	15.597	13.599	11.513

**Table 7:** MAPE of six types of air pollutant concentrations between self-built points, model forecast values and national control point.

Input variable	Self-built points	SRA	MLP	SRA-MLP
PM2.5	0.447	0.166	0.176	0.163
PM10	0.887	0.221	0.213	0.205
CO	0.478	0.313	0.233	0.183
NO_2_	2.129	0.554	0.398	0.287
SO_2_	0.685	0.656	0.441	0.394
O_3_	4.322	1.124	0.985	0.635

It can be seen that whether it is a stepwise regression model, or the MLP and SRA-MLP models, the prediction accuracy is better than the measurement accuracy of self-built points. This shows that using the three established mathematical models to calibrate the measurement data of self-built points can achieve better results. Since the error evaluation index of the SRA-MLP model is the smallest among the three models, the SRA-MLP model is selected to calibrate the measurement data of self-built points. Among the six types of pollutant prediction models, the accuracy of the PM10 prediction model's RMSE has the largest increase, with an accuracy increase of 74.4%. The PM10 prediction model's MAE has the largest increase in accuracy, with an accuracy increase of 76.3%. The NO_2_ prediction model's MAPE has the largest increase in accuracy, with an accuracy increase of 86.5%.

**Figure 7:**
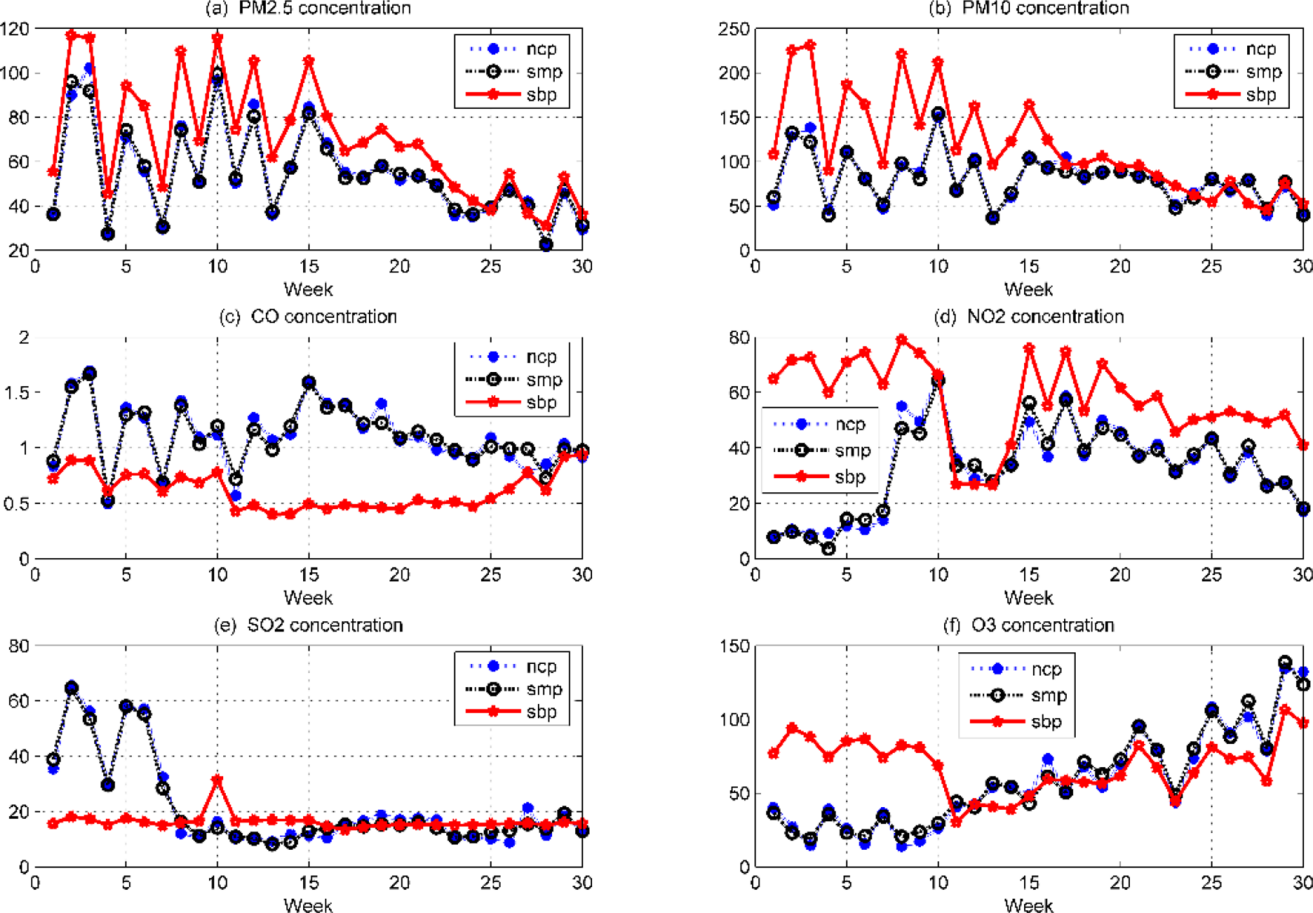
Comparison of weekly average data of six types of pollutants at national control points, self-built points and SRM-MLP model correction points.

The concentration of pollutants in the atmosphere has an obvious correlation with the periodic activities of human beings. The weekly averages of the six pollutant concentrations are plotted in Fig. 7. It can be seen that there is a significant deviation between the red self-built point data curve and the blue national control point data curve, but the black model fitting value (smp) curve deviates very little from the national control point data curve. The results show that the accuracy of the SRA-MLP model for predicting the concentration of pollutants is better than the accuracy of the self-built point measurement data.

## Conclusions

The air quality index (AQI) is a dimensionless index that quantitatively describes the condition of air quality. It is often used to measure the quality of air quality. The main pollutants participating in the air quality assessment are PM2.5, PM10, CO, NO_2_, SO_2_, O_3_, etc. Therefore, to realize the monitoring of air quality, it is very important to monitor the concentration of ''two dusts and four gases" in real time.

Many countries have established national monitoring and control stations to monitor air pollutant concentrations. Although the national control point is more accurate in monitoring pollutants, the cost of deployment is high, the number of deployments is small, and the maintenance costs are high. Therefore, it is difficult for the national control point to achieve full control. The miniature air quality detector developed by some companies has successfully improved these shortcomings, but the accuracy of monitoring needs to be improved.

The pollutant correction model based on the stepwise regression model has some corrections to the self-built point data, and the results obtained are easier to interpret, but the correction effect needs to be improved. Compared with regression models, artificial neural networks have a greater advantage in data correction. The artificial neural network does not rely on the typical distribution of the original data. It simulates human thinking to derive a non-linear mapping relationship between the input and output of the system, and then makes intelligent reasoning and prediction.

The SRA-MLP model given in this article combines the advantages of a stepwise regression model and an artificial neural network combined model. It not only provides the quantitative relationship between the monitoring data of self-built points and the concentration of the six pollutants, but also greatly improves the accuracy of the prediction of the concentration of the six pollutants. The data used in the model is 4135 groups, the time span is 206 days, and the data of all four seasons are involved, and it shows good predictive ability in the training set and the test set, so the model is very stable. This model plays a positive role in grid-based monitoring of the concentration of various pollutants and guides the scientific deployment of miniature air quality detectors. It can also be popularized and applied to the prediction of environmental pollution indexes such as water pollution, soil pollution, noise pollution and light pollution. But because this research uses a small data set, it is not suitable for deep learning. In future research, we hope to collect more data and use deep learning to improve the model.
